# The Management of Erythrodermic Psoriasis Complicated by Cyclosporine

**DOI:** 10.1155/2020/5215478

**Published:** 2020-09-08

**Authors:** Suman Rao, Michelle Bernshteyn, Raman Sohal, Rachael Proumen, Alexandra Goodman, Zachary Shepherd

**Affiliations:** SUNY Upstate Medical University Hospital, 750 E Adams St., Syracuse, NY 13210, USA

## Abstract

We present a 64-year-old woman with past medical history of psoriasis and alcoholic liver cirrhosis who presented with a diffuse, erythematous, and scaly rash. Pertinent medications included topical triamcinolone 0.1% cream. She was started on oral prednisone 40 milligrams (mg) and oral cyclosporine 150 mg daily and was continued on topical triamcinolone. After the administration of two doses of this regimen, the serum creatinine increased to 1.76 mg/dL, and serum potassium increased to 6.7 mEq/L. The serum creatinine continued to uptrend to 2.42 mg/dL, and the glomerular filtration rate (GFR) decreased to 20 mL/min. The patient was emergently hemodialyzed. The patient was placed on an extended steroid taper, alleviating the psoriatic rash. However, the patient needed to be placed on a steroid-sparing regimen. Because of its rarity and ensuing complications, erythrodermic psoriasis must be identified and managed promptly. Cyclosporine is currently the first-line treatment. However, initiation of this therapy in our patient resulted in an acute kidney injury (AKI). Even though a steroid taper assisted in alleviating erythroderma, a steroid-sparing regimen needed to be started. This led to the consideration of alternate methods of therapy for further management of erythrodermic psoriasis with renal impairment.

## 1. Introduction

Psoriasis affects 3% of the adult population in the US. However, erythrodermic psoriasis is the rarest subtype, manifesting in less than 3% of patients with psoriasis [1]. Because of its rarity and capability to become life-threatening, the identification and appropriate treatment of erythrodermic psoriasis is essential. Erythrodermic psoriasis typically presents with erythema encompassing more than 75% of the skin surface [2]. It can be associated with pustules, scaling, or exfoliation. Although there is a scarcity of epidemiologically relevant data, erythrodermic psoriasis has a preference for manifestation in men and an average age of presentation at 48. Even though the immunopathogenesis of erythrodermic psoriasis is not fully understood, studies have shown it to be similar to that of plaque psoriasis, related to differential expression of the interleukin 17A signaling pathway [3]. Risk factors for the development of erythrodermic psoriasis include the administration of systemic glucocorticoids, abrupt withdrawal of either methotrexate/cyclosporine, treatment with topical retinoids, overuse of topical steroids, or TNF alpha inhibitors [2]. Complications of erythrodermic psoriasis include increased frequency of infections, including sepsis, due to breakdown of the skin defense barrier, and electrolyte abnormalities due to loss of fluid [4].

Cyclosporine is the treatment of choice for patients with erythrodermic psoriasis, even though the efficacy of this medication is limited [5]. The adverse side effects of this medication, specifically nephrotoxicity, have mainly been studied in patients undergoing kidney, liver, or heart transplants [6]. Chronic cyclosporine-induced nephrotoxicity can be seen in the form of chronic kidney disease and electrolyte/acid-base disturbances such as hyperkalemia, hyperuricemia, or gout [7, 8]. The pathogenesis of chronic nephropathy is secondary to renal insufficiency due to glomerular and vascular disease, tubular function abnormalities, and hypertension. Acute cyclosporine-induced nephrotoxicity can be characterized by AKI secondary to vasoconstriction of the afferent and efferent arterioles and a subsequent decrease in renal blood flow and GFR [8].

## 2. Case Presentation

We present a 64-year-old woman with past medical history of poorly controlled psoriasis and alcoholic liver cirrhosis who presented with a diffuse, erythematous, and scaly rash and desquamation for three weeks, worsening over the past week. She also complained of progressive weakness, subjective fevers, dyspnea at rest, and bilateral lower extremity swelling. She was in excruciating pain, tearful, and had intense pruritus. This patient had a diffuse, erythematous, and scaly rash and large plaques on her scalp, chest, abdomen, arms, and legs, which are the clinical presenting signs of erythrodermic psoriasis. She had a previous skin biopsy which confirmed her diagnosis of psoriasis as well.

Physical examination was remarkable for tachycardia at 115 beats per minute and new-onset hypoxia requiring 4 liters of oxygen by nasal cannula. Skin examination revealed large psoriatic plaques on her scalp, chest, abdomen, arms, and legs, along with diffuse erythroderma. The skin was dry, thin, and tender to palpation. The dorsal aspect of the left leg showed a weeping ulceration with serosanguinous discharge. 3+ bilateral lower extremity pitting edema was also present. Labs on admission showed a white blood cell count of 11,600 cell/mm^3^, hemoglobin of 12.1 g/dL, hematocrit of 36.5%, sodium of 122 mEq/L, potassium of 5.8 mEq/L, chloride of 89 mEq/L, bicarbonate of 21 mEq/L, blood urea nitrogen of 22 mEq/L, and creatinine of 1.95 mg/dL (with a baseline creatinine 0.9 mg/dL). GFR was 26 mL/min. Chest X-ray on admission showed atelectasis of the left lower lobe, and electrocardiogram showed sinus tachycardia.

Past surgical history and family history were noncontributory. Social history included former smoking at one pack per day, which she quit ten years prior to admission, and her last alcoholic drink was three months prior to admission. She did not qualify for the liver transplant list due to extent of alcohol use and relapses. Pertinent medication history included topical triamcinolone 0.1% ointment, without any systemic steroidal therapy. Other medication history included diuretics (furosemide and spironolactone), naproxen, lactulose, baclofen, ranitidine, and potassium supplements. She was treated with apremilast two years prior with significant improvement in psoriatic plaques. However, due to worsening hepatic function and underlying cirrhosis, apremilast was discontinued a year prior to presentation.

The patient denied chest pain, cough, vomiting, or diarrhea. She denied having chills, night sweats, or weight loss. The patient did not have signs of onycholysis or arthritis. She has no prior history of chronic kidney disease or recent history of infections.

After admission, the electrolyte abnormalities and AKI were the first two problems that were managed. Hyperkalemia was treated with insulin, furosemide, Kayexalate, and lactulose. Asymptomatic hyponatremia and AKI were treated with normal saline. After this regimen, the serum creatinine began trending down toward normal at 1.67 mg/dL a day after admission. GFR began to uptrend to 31 mL/min.

Dermatology was consulted and recommended starting oral prednisone 40 mg daily and oral cyclosporine 150 mg daily. They also recommended oral antihistamines for pruritus, topical emollients for moisturization, and continuing topical triamcinolone.

After the administration of two doses of this regimen, the serum creatinine began to uptrend to 1.76 mg/dL, and the potassium level increased to 6.7 mEq/L. Cyclosporine was discontinued at this time. The serum creatinine continued to uptrend to 2.42 mg/dL on the following day. GFR continued decreasing to 20 mL/min. The patient was sent for vascular catheter placement and emergent hemodialysis, after a serum potassium level of 6.7 mEq/L.

After completing hemodialysis, her lab work showed serum sodium of 126 mEq/L, potassium of 4.4 mEq/L, chloride of 91 mEq/L, bicarbonate of 22 mEq/L, blood urea nitrogen of 16 mEq/L, creatinine of 1.58 mg/dL, and GFR of 34 mL/min. On the sixth day after admission, her lab work showed serum sodium of 128 mEq/L, potassium of 4.0 mEq/L, chloride of 95 mEq/L, bicarbonate of 24 mEq/L, blood urea nitrogen of 19 mEq/L, creatinine of 0.73 mg/dL, and GFR of 89 mL/min.

The patient was continued exclusively on extended steroid taper. The generalized psoriatic rash appeared significantly decreased. However, the patient needed to be placed on a steroid-sparing regimen.

## 3. Discussion

### 3.1. Approach to the Initial Management of Erythrodermic Psoriasis

This patient was previously diagnosed with psoriasis confirmed on skin biopsy, presenting with the tell-tale signs of this disease. The progression toward erythrodermic psoriasis in this patient was likely the result of poorly managed psoriasis. Her only treatment on admission was topical triamcinolone 0.1% cream. Studies have shown that excessive use of topical triamcinolone alone can trigger the manifestations of erythrodermic psoriasis. In addition, this patient was not on any systemic antipsoriatic medications after the discontinuation of apremilast two years ago, which could have contributed to her current presentation. During her admission, a decision was made against restarting apremilast due to underlying cirrhosis.

Cyclosporine is currently the first-line treatment for erythrodermic psoriasis, even though data on the efficacy of this drug for this specific disease subtype are lacking [5].

### 3.2. Acute Cyclosporine-Induced Nephropathy

After the initiation of cyclosporine, the patient experienced AKI, with the serum creatinine trending upward to 1.76 mg/dL and the potassium level to 6.7 mEq/L. After discontinuation of cyclosporine, the serum creatinine continued to trend upward to 2.42 mg/dL on the following day, attributable to cyclosporine's half-life of 8.4 hours. Observably, the only two medications started prior to the AKI were the steroid taper and the cyclosporine. Since steroids do not have any documentation of causing nephrotoxic symptoms, we propose that the cyclosporine was the cause of her ensuing AKI.

Although there have been several studies that demonstrate the nature of cyclosporine-induced nephropathy, there are distinct aspects of this presentation that are intriguing. More commonly, the nephropathy secondary to cyclosporine occurs after a prolonged exposure with chronic use. The onset of this patient's nephrotoxicity was within a couple of hours after the administration of cyclosporine and persisted until the completion of emergent hemodialysis. Although acute nephrotoxicity secondary to cyclosporine has been studied, most of these studies have been in postkidney transplant patients [6].

### 3.3. Alternative Methods of Therapy

After the discontinuation of cyclosporine, the patient was continued on an extended steroid taper. She seemed to experience alleviation of her symptoms and a decrease in her psoriatic rash. However, she needed to be placed on a steroid-sparing regimen, and alternative methods of therapy were explored.

Studies have shown that infliximab is another first-line of therapy for erythrodermic psoriasis [9]. Other alternative therapies such as acitretin and methotrexate are less studied but are known to have a slower onset of action. Adalimumab and ustekinumab have also been useful in treating patients with moderate to severe plaque psoriasis [10].

In patients with acute renal failure or chronic kidney disease, alternative pharmacological agents must be considered in order to ensure an efficacious treatment, with minimal side effects. Studies have shown that methotrexate as well as apremilast should be avoided in patients with psoriasis [11]. In patients with decreased renal function, etanercept has been shown to be tolerated well in the management of psoriasis. Leflunomide has also been an appropriate alternative [12]. Other studies have shown that adalimumab can be safely used in patients presenting with end-stage renal disease, requiring hemodialysis [13].

## 4. Conclusion

Erythrodermic psoriasis manifests in less than 3% of all patients with psoriasis, presenting with scaly, erythematous rash, desquamation, and exfoliation involving more than three-fourths of the skin surface area. Because of its rarity and the ensuing complications, identifying and managing erythrodermic psoriasis is highly important. Cyclosporine is currently considered the first-line treatment for erythrodermic psoriasis. However, initiation of this therapy in our patient resulted in acute presentation of AKI. Although discontinuation of cyclosporine and initiation of a steroid taper assisted in resolving her AKI and alleviating the erythroderma, a steroid-sparing regimen that was compatible with her renal issues needed to be added to the management of this patient.
